# Minority College Women’s Views on Condom Negotiation

**DOI:** 10.3390/ijerph13010040

**Published:** 2015-12-22

**Authors:** TyWanda McLaurin-Jones, Maudry-Beverly Lashley, Vanessa Marshall

**Affiliations:** 1 Department of Community & Family Medicine, Howard University College of Medicine, 520 W Street, NW, Washington, DC 20059, USA; vmarshallv44@gmail.com; 2 Department of Psychology, Medgar Evers College, 1650 Bedford Avenue, Brooklyn, NY 11235, USA; mlashley@mec.cuny.edu

**Keywords:** minority college women, condom use, sexually transmitted infections, sexual behavior, negotiation strategies

## Abstract

This study utilized quantitative and qualitative methods to (1) investigate the relationship between frequency of condom use and negotiation strategies and (2) evaluate experiences with condom negotiations among sexually active, heterosexual, African American college women. One hundred female students from a Historically Black Colleges and Universities (HBCU) completed a questionnaire that included the Condom Influence Strategies Scale (CIS) and participated in a focus group. An ANOVA was conducted to compare differences between never, inconsistent, and consistent condom users. Consistent condom users scored higher than never users on the “withholding sex” subscale of the CIS (4.88 *vs.* 3.55; *p* < 0.001) as well as endorsed items more strongly on the “direct request” subscale of the CIS (4.63 *vs.* 3.82, *p* < 0.05) than never users. A thematic analysis of open discussions identified overarching themes. Similarly, refusing sex and/or having direct communications with partner emerged as primary strategies. Threats to negotiation included deciding the “right timing” of discussion and having a previous history of sexual intercourse without a condom with their partner. Other key concepts that contribute to condom negotiation are the views that condoms are a male’s responsibility and stigma of women who carry condoms.

## 1. Introduction

Sexually transmitted infections (STIs, which include, but not limited to, chlamydia, gonorrhea, syphilis, human papillomavirus infection, and genital herpes) in the United States (US) remain a major public health concern, with a prevalence of 110 million and 20 million new cases every year [1,2]. Adolescents, young adults, women, and ethnic minorities are particularly vulnerable to STIs. The youth (15–24 years) acquire about 50% of all new infections; the rate of chlamydia is 2 times greater in women than men and 6.4 times greater in African Americans (AA) than Caucasian Americans (CA), while gonorrhea is 12.4 times greater in AAs than CAs [1,2]. Hence, being a young AA woman may pose the greatest risk, as young AA women have the highest prevalence rates across a variety of STIs [1,2].

Environmental factors such as a collegiate atmosphere may also play a major role in the increase rates of STIs. Attending college is a critical junction for young adults because they are shifting from a role of parental dependence to one of independence, particularly those who may be experiencing their first time away from home. With this new found independence, comes responsibilities that students may find challenging. One such challenge revolves around the shaping of their behaviors and most importantly, their sexual well-beings. Because college students are shaping their sexual behaviors, they may be more likely to engage in risky sexual behaviors, such as multiple partners, serial monogamy, high number of relationship partnerships, and alcohol and drug use [3,4]. Students’ engagement in the aforementioned behaviors may be an attempt to gain a variety of experiences before committing to long-term relationships, but such behaviors place them at greater risks for acquiring STIs [3,5]. This is of particular significance given that AA college women specifically are more likely to be infected with HIV/AIDS while in college and attending a HBCU is associated with higher rates of STIs [6,7].

Although condom use remains an important preventive health behavior, college students have a low percentage of consistent condom use [5,8,9,10]. AA women have been found to be less likely to report condom use and the rate of “always use a condom” is about 24 percent [8,11]. To understand the low rates, researchers have investigated a variety of intrapersonal and interpersonal factors. Research has demonstrated that AA college women have adequate knowledge of the STI/HIV transmission process, self-efficacy and confidence to use condoms, assertiveness, and the communication skills necessary to negotiate condom use; yet condom use remains low [12,13,14,15,16].

Women communicating condom use to their heterosexual partners may be dependent on several factors including anticipation of negative attitudes towards condom use from the male partner; whether the relationship is a committed or causal one; their personal attitudes towards condom use, and the perception of condom use among female peers [6,11,17]. Culturally appropriate and gender-based directives for sexual health promotion and condom negotiation are needed. Hence, it is important for researchers and health educators to examine how AA college women facilitate discussions about condom use to ascertain strategies for safer sex practices. Furthermore, in a community sample of ethnically diverse adult men and women, Noar and colleagues demonstrated a positive relationship between frequency of condom use and specific condom negotiation strategies [18]. A similar investigation of AA college women may provide insight in to this phenomenon.

Therefore, the objective of this study is two-fold. First, we investigated the relationship between condom negotiation strategies and frequency of condom use among college women of African descent. Secondly, we sought a deeper understanding of the experiences women had discussing condom use with their partners.

## 2. Experimental Section

### 2.1. Study Design

Mixed methods, including surveys and focus groups, were conducted to evaluate young adult women of African descent attending an HBCU. Guided by the concurrent procedural strategy, a cross sectional study design was used to examine the frequency of condom use and condom negotiation strategies [19]. To add a richer meaning to the quantitative data, an open discussion of condom use via focus groups was guided by the phenomenological approach. The study protocol was approved by the university’s Institutional Review Board.

### 2.2. Sampling Procedures

To assess young adult women of African descent in a college setting and exclude individuals who do not suit the objective of this study, the sample was a purposive sample of convenience. Sample size was based on DePaulo’s recommendation of 100 participants to ensure that the widest range of perceptions and opinions were accounted and represented [20]. Thus, 100 women were recruited from an undergraduate program on the East Coast via posting/distribution of flyers and participant pool e-mail notifications. Potential participants contacted study researchers and were given a confidential screen to determine eligibility. Individuals were invited to participate if they were (1) of African descent; (2) enrolled in college at least part-time; (3) between the ages of 18 and 24; (4) identified as heterosexual; and (5) were sexually active within the last 90 days.

### 2.3. Study Procedures

Students were scheduled for 3 h group sessions held on pre-selected dates arranged in the evenings and weekends to accommodate their schedules. Group membership was based on students’ availability of date and time. A total of 13 sessions were held with an average of 7.69 college women per group. On the day of study participation, participants reported to a conference room and were seated around a rectangular table with the lead author (TMJ) sitting at the head. Light refreshments were available at a separate table. After obtaining written informed consent by the lead author, participants completed a pencil-and-paper questionnaire (approximately 45 min in length) with a pre-assigned identification number to protect their identity.

Upon completion and collection of all of the surveys, the focus group was convened by the lead author who re-introduced herself, stated the purpose of the focus group, and encouraged participants to share their experiences, values, and preferences about a variety of topics related to condom use. Prior to the discussion, participants were instructed to choose a “pseudo-name” and to write that name on a tent card which was placed in front of the participant. Additionally, participants were requested to “not share” any information from the focus group with family or friends to further safeguard participants’ confidentiality. A structured interview open-ended format was qualitatively utilized. Each focus group was conducted by using a common guide with the same questions in order to ensure standardization across the focus groups. For participants who were hesitant in their responses, the lead researcher was able to encourage them to actively engage by calling on them and soliciting their views. The moderator, who also attended an HBCU, is of African descent thereby reducing any threat to the participants [21]. All group discussions were audiotaped using two digital recorders and lasted approximately two hours each. Every participant received a $50 visa gift card as reimbursement for her time.

### 2.4. Measures

To obtain general characteristics of the sample, participants’ completed a demographic questionnaire. Items included age, sub-cultural identity (American, African, Caribbean, or Bicultural), level of education (freshman, sophomore, junior, senior), housing status (on campus or off campus), employment (employed or not employed), and grade point average (GPA) on a four point scale.

Overall sexual health included a seven-itemed questionnaire. Age of sexual initiation was measured by an open-ended question, “How old were you when you first had consensual vaginal intercourse with a male”. Current relationship status was measured by “which of the following best describes your current relationship status?” with three corresponding choices, “exclusive monogamous”, “non-exclusive/non-monogamous”, or “not in a relationship”. Participants were also asked to indicate the total number of monogamous and non-monogamous relationships as well as indicate whether or not they have been tested for HIV (yes/no) and or if they have been treated for a STI (yes/or no). Finally frequency of condom use was measured by a single item: “How often do you use condoms with vaginal sex?” with corresponding response choices, “never”, “sometimes”, “most of the time”, and “always”.

Women’s condom negotiation was measured by the Condom Influence Strategy Questionnaire (CISQ). The CISQ contains 36 strategies in which the respondent is asked to think about her current/most recent intimate partner and rate the likelihood from very likely “1” to very unlikely “5” that she would use the strategy to ensure that a condom is used during sexual activity, even if she has not engaged in the activity [22]. Ratings are reverse scored so that higher scores indicate higher endorsement of the strategy. Ratings are summed to yield six subscales: Withholding Sex (e.g., “Let my partner know that no condom means no sex”), Direct Request (e.g., “Ask that we use condoms during sex”), Seduction (e.g., “Take out a condom to use without saying a word”), Relationship Conceptualization (e.g., “Tell my partner that since we love and trust one another that we should use a condom”), Risk Information (e.g., “Tell my partner that using a condom will protect us from STDs”), and Deception (e.g., “Tell my partner that we should use a condom to prevent pregnancy even though my real worry is STDs”). The CISQ was found to be highly reliable for the current sample (Withholding Sex α = 0.94; Direct Request α = 0.94; Seduction α = 0.92; Relationship Conceptualization α = 0.94; Risk Information α = 0.94; Deception α = 0.83).

To further evaluate college women’s negotiation strategies, focus groups were used to capture participants’ experiences and views which may not have been developed or stimulated by quantitative measures. Focus groups as an aspect of qualitative research methods allow individuals to have an open, detailed, in-depth dialogue and articulate broader views on topics, such as condom use, not openly discussed by the larger society [23]. Alternatively, focus groups allow for deindividuation as such individuals may not feel comfortable in expressing these views in a one-on-one interview [24]. The focus group interview guide was developed in advance and was designed to examine how the nature and context of college relationships, perception of risk, and substance use influenced condom use. The guide also elicited women’s preferences for campus-based interventions. The interview questions reported here represent a subset of a larger project on condom use. Participants were specifically asked the following from the guide: “How do/did you discuss condom use with your partner?” and “What concerns do you have about discussing condom use with your partner or potential partner?” The last question, “Who is responsible for the condom?” arose from the first focus group. The question was added to the interview guide so that each group received the same questions.

### 2.5. Data Analysis

Quantitative data analyses were conducted using IBM SPSS Statistics (version 22.0, SPSS Inc., Chicago, IL, USA). First, participants were classified into three groups according to frequency of condom use with vaginal sex. Women who self-reported “always” using a condom were coded as consistent condom users (CCUs). Women who reported “sometimes” or “most of the time” were collapsed and coded as inconsistent condom users (ICUs). Finally, women who reported “never” using a condom were coded as non-condom users (NCUs). This coding strategy resulted in 31 CCUs, 56 ICUs, and 13 NCUs. Chi-squares and ANOVAs (as appropriate) were conducted to compare NCUs, ICUs, and CCUs on demographics, sexual health, and the six subscales of the CISQ.

All focus group recordings were transcribed verbatim by an independent transcription company. Accuracy of the transcripts was verified against the original recording by all authors (T.M.J., M.L., V.M.). Participants were allowed access to their focus groups’ transcripts and given the opportunity to provide feedback to establish credibility of the data. Upon verification, transcripts were analyzed using inductive thematic analysis based on the work of Braun and Clark [25]. Each author reviewed a subset of focus group transcripts and developed an individual preliminary list of codes. These individual lists were compared and refined through team discussion. The initial round of analysis assessed the specific questions with preliminary codes applied. The secondary and tertiary rounds of analyses were used to reduce the data into themes. Various data analysis strategies were used, including identifying codes, the process of reading, rereading, questioning, and synthesizing the data to establish triangulation [19]. Through discussions and iterative review of codes and themes, consensus was reached. Any discrepancies between the researchers were resolved through discussion [26]. The thematic analyses were based on patterns of the responses that emerged. Trustworthiness was established by looking for depth and detail, vivid and nuanced answers that were rich with themes.

## 3. Results & Discussion

### 3.1. Frequency of Condom Use and Sample Characteristics

Overall college women were approximately 20.42 years old and had an average 3.18 GPA. Approximately 74% described themselves as African American and the sample contained more seniors (37%) than any other collegiate level. There were no significant differences regarding demographic characteristics by condom group membership (Table 1). In terms of sexual health (Table 2), college women reported an average sexual initiation age of 16.66 years. This is comparable to both Hou and Valentine *et al.* who found a sexual debut age of 16 years [7,27]. Women reported a history of about 4 (average of 2 monogamous and 2 non-monogamous) sexual relationships. This finding is slightly higher than the average number of 3 partners found in the literature [28].

**Table 1:** Frequency of condom use and demographic characteristics of minority college women.

Characteristic	Total *n* = 100	Non Condom Use *n* = 13	Inconsistent Condom Use *n* = 56	Consistent Condom Use *n* = 31	Statistic	*p*
Age (M ± SD)	20.43 ± 1.20 years	20.83 ± 1.19 years	20.46 ± 1.19 years	20.20 ± 1.21 years	F = 1.255	0.290
GPA (M ± SD)	3.18 ± 0.40	3.12 ± 0.37	3.17 ± 0.42	3.20 ± 0.37	F = 0.146	0.864
**Ethnic Identity**						
African American	74%	84.4%	73.2%	71.0%	χ^2^ = 1.574	0.813
African National	7%	0.0%	7.1%	9.7%		
Caribbean	19%	15.6%	19.6%	19.4%		
**College Year**						
Freshman	10%	0.0%	12.5%	9.7%	χ^2^ = 6.853	0.335
Sophomore	24%	15.4%	21.4%	32.3%		
Junior	29%	30.8%	25.0%	35.5%		
Senior	37%	53.8%	41.1%	22.6%		
**Campus Housing**	59%	46.2%	53.6%	74.2%	χ^2^ = 4.527	0.104
**Employment**	45%	53.8%	44.6%	41.9%	χ^2^ = 0.532	0.767

M = Mean; SD = Standard Deviation.

A large percent of the sample reported having been tested for HIV (88%), while more than a third reported a history of a STI. The majority of the women (50%) reported currently being in a monogamous sexual relationship. This was the only sexual history characteristic that differed significantly by condom group. NCUs were more likely to report currently being involved in a monogamous relationship, whereas, CCUs were more likely to report “not in a sexual relationship” (*p* = 0.040). This is consistent with the research as demonstrated that AA college students involved in monogamous relationships are less likely to use condoms [29].

**Table 2:** Frequency of condom use and sexual history characteristics of minority college women.

Characteristic	Total *n* = 100	Non Condom Use *n* = 13	Inconsistent Condom Use *n* = 56	Consistent Condom Use *n* = 31	Statistic	*p*
**Age of Initiation (M ± SD)**	16.66 ± 1.80 years	16.15 ± 2.15 years	16.50 ± 1.72 years	17.16 ± 1.73 years	F = 1.978	0.144
**Sexual Relationships**						
Monogamous	2.11 ± 1.50	2.69 ± 1.97	2.25 ± 1.48	1.61 ± 1.20	F = 3.032	0.053
Non-Monogamous	2.01 ± 2.81	2.85 ± 2.85	2.16 ± 3.09	1.39 ± 2.17	F = 1.426	0.245
**Relationship Status**						
Monogamous	50%	84.6%	50.0%	35.5%	χ^2^ = 10.049	0.040
Non-monogamous	14%	7.7%	16.1%	12.9%		
No Sexual Relationship	36%	7.7%	33.9%	51.6%		
**Tested for HIV**	88%	84.6%	94.6%	77.4%	χ^2^ = 5.768	0.056
**STI History**	35%	30.8%	44.6%	19.4%	χ^2^ = 5.726	0.057

M = Mean; SD = Standard Deviation.

### 3.2. Frequency of Condom Use and Condom Influence Strategies

As evidenced in Table 3, minority collegiate women endorsed withholding sex (M = 4.31) and making a direct request (M = 4.27) negotiation strategies to a greater degree than seduction (M = 3.32), relationship conceptualization (M = 3.34), risk information (M = 3.50) or deception (M = 2.54). This pattern of responding is similar to Holland and French’s sample that included men and women [5]. Furthermore, the withholding sex and the direct request strategies differed significantly by condom group. Specifically, CCUs endorsed items more strongly than NCUs on the withholding sex subscale (4.88 *vs.* 3.55; *p* < 0.001) and the direct request subscale (4.63 *vs.* 3.82, *p* < 0.05) of the CISQ. This finding is similar to Noar, Morokoff, and Harlow who also found a significant positive correlation between frequency of condom use and condom influence strategies [18].

**Table 3:** Frequency of condom use and condom influence strategies of minority college women.

Condom Influence Strategy	Total *n* = 100		No Condom Use *n* = 13		Inconsistent Condom Use *n* = 56		Consistent Condom Use *n* = 31		F	*p*
	M	SD	M	SD	M	SD	M	SD		
Withhold Sex	4.31	1.03	3.55 ^a^	1.47	4.17 ^a,b^	1.03	4.88 ^b^	0.26	10.51	0.000
Direct Request	4.27	1.06	3.82 ^a^	1.31	4.17 ^a,b^	1.06	4.63 ^b^	0.85	3.37	0.038
Seduction	3.32	1.32	2.61	1.39	3.34	1.41	3.59	1.03	2.56	0.082
Relationship Conceptualization	3.34	1.37	2.89	1.55	3.33	1.39	3.54	1.23	1.05	0.356
Risk Information	3.50	1.38	3.06	1.52	3.41	1.30	3.84	1.45	1.72	0.185
Deception	2.54	1.14	2.74	1.28	2.62	1.16	2.31	1.05	0.96	0.385

M = Mean; SD = Standard Deviation. ^a,b^ Groups with same superscript letter indicates no group differences. Groups with different superscript letter denotes group differences.

### 3.3. Condom Discussions

An overwhelming number of participants equated not using a condom with a death wish. Protection was paramount in their relationships. For example, one participant was able to capture this belief in the following quote:

“It’s almost like to me unprotected sex is like you having a gun and you shooting it at me and you just shoot the gun and I don’t have no bulletproof vest on so you shooting at me like I’m just your practice thing, you know?”

Overall, responses from the women in the study indicated the importance of having a conversation around condom use. There was a wide consensus that it is important to begin the discussion of condom use early in the relationship so that such issues could be readily addressed before having sex. Three primary types of encounters emerged.

#### 3.3.1. Theme: Direct Communication

In discussing condom use a majority of participants shared that there must always be an open, honest and direct dialogue with their partners/potential partners. One participant captured the importance of direct communication as a means of self-confidence and shared:

“Just having confidence in yourself, and knowing that if I’m direct about it now, it’s going to help me in the long run, because if you’re confident in addressing the issue, you’ll be confident in walking away from a situation where someone disagrees with you about condom use.”

In addition, several participants acknowledged that maturity enabled them to directly address this issue with their partners. This was expressed by the following participant:

“Sex is about maturity. And when you are mature enough to go to the store and walk up to that cash register and have them ring up the condoms or spermicide or whatever, then you’re ready for sex. But before then, you need to seriously take a look at yourself and say, is this something that I’m truly ready for?”

#### 3.3.2. Theme: No Condom then No Sex

Many participants expected their male partner to have the condom and would not follow through with expected sexual intercourse if a condom was not present. One female participant observed that,

“For me it was just always an expectation that was just kind of like in the air, like there was never a certain conversation where I was like, ‘Oh, do you use condoms?’ or things like that. It was just always expected, like and if you—if you’re planning on not using the condom, especially the first time it’s just not—like we’re just not having sex. You can leave.”

Another female participant recounted a conversation that she had with a male partner:

“Do you have a condom? Oh you don’t? Oops. Guess we’re just going to go back to doing what we were doing before then because it’s not going to occur.”

Participants also discussed their behavioral reactions to not having access to a condom as illustrated by a participant.

“I’ve literally had to like roll off the bed, like, and have my escape plan. I tell people something once, if you don’t want to listen that one time I’m not even giving you the second chance.”

#### 3.3.3. Theme: Education on Risk

The third type of discussion encountered by AA college women involved education on risk. The issue of discussing the importance of educating their partners on condom use occurred in all groups. Participants own comfort level was evident as they addressed their own concerns with their partners. Several of these women shared that they provided their partners with visual aids including using diagrams and watching educational videos in an effort to familiarize their partners on the importance of using condoms.

“I always break it down to him like I’m talking to my students and stuff like that, (like with my current boyfriend, like) when we first started talking about it I’m like we need to use condoms (and this and that) because this so and so could happen. And he thought it was kind of funny because like I actually pulled out my diagrams and stuff that can happen if we didn’t use one and he was like, ‘What is that? What can you get from that?’ I was like, ‘You can catch this. You can do that from touching’, like I just broke everything down and he was like, “Oh okay”. He’s like, “You’re on birth control?” I was like, “Yeah I am”, but I had to explain to him and he was like, “What type are you on?” I just broke everything down to him like I was teaching a class, basically, and that’s kind of how I have always been.”

Some participants shared that some of the men had taken responsibility for educating their partners and had videos playing on the importance of having protected sex in an effort to reduce STIs whenever their female partners visited.

“A friend of mine and her significant other was dealing with each other and they was just like talking. He was watching a series that is used over there to like help combat STDs and what-not. He basically had it playing, um, when she went to his room. So that’s how they went about having protected sex.”

These findings illustrate that AA college women have a major role to play in condom negotiations with the majority of college women highlighting the importance of negotiation strategies. Roundtree & Mulraney suggest that when discussing condom use among AA women, it is important to take into consideration cultural experiences which may influence some women in not being assertive in condom negotiations [30]. Previous research suggests that the male-to-female ratio at HBCUs lead to women complying with men’s condom use preferences [6]. The current sample was strong in their convictions about condom use and the protection that was provided. Women were willing to walk away from situations when their protection needs were not met. In addition, several of the women in this study felt that they had to be prepared for disagreements particularly from their partners if they did not want to use a condom. In such a case, a number of women in our study suggested that they had to be direct and educate their partners on the benefits of condom use. Findings exemplify participants’ progressive views and the use of rational strategies in condom negotiations. However, only a third of the women reported consistent condom use. The difference in the strength of convictions/use of rational strategies reported in the focus groups and their actual condom use may be attributed to how well each female negotiates with her partner and if other risky behaviors, such as alcohol or marijuana, are involved at the time of sexual intimacy.

### 3.4. Concerns about Condom Discussions

While women in the sample emphasized the importance of condom use and the desire to protect themselves and their partners, they acknowledge the difficulty of having condom discussions. Two major themes emerged.

#### 3.4.1. Theme: Timing-Appropriate Time to Discuss Condom Use

The issue of timing was a major theme among discussants. Several participants felt that the time to discuss condom use should occur in the initial phase of the relationship, which provided an opportunity to negotiate condoms prior to sexual intimacy. Yet, participants acknowledge the difficulty in bringing up condom use and how it may influence the way their potential partner thinks about their character.

“So I think that it’s kind-of hard to jump from that oh hey, you’re cute, what’s your favorite color, what you like to do, so when we get to this point, are you going to use condoms, like she said, it’s kind-of a little bit presumptuous because then he’s one, going to think you want it now, or he’s going to shy away and be like, Oh, she’s one of those chicks, let me move on to the next one.”

#### 3.4.2. Theme: History of Unprotected Sex with Partner

Participants across all groups admitted that a decision not to use a condom in the initial phase of their relationship presented major problems when they sought to use a condom at a later time. Some participants highlighted issues of fidelity which became challenging for them. Several participants highlighted the issue of cheating:

“It just would make him question me. Or it’s like, ‘Do you think I’ve been cheating on you? Why all of a sudden? We’ve been having sex without a condom for this long? Why all of a sudden do you want to use a condom now?’ ”

“And so he found a bag of condoms in my purse and he’s like, ‘What’s this about? You know we don’t use condoms but you have a bag of, uh, a lot of condoms in your purse. Okay explain this to me now.’ ”

Other participants did not want to have hurt feelings or to cause hurt feelings. One female student recounted her conversation which echoed other sentiments:

“...he found it difficult because you’re going to say, you know, well people are telling me oh I need to protect myself but we started off and established a relationship when we’re not using so now how do I say, ‘Let’s use’ because everyone's saying it’s—it’s the right thing to do, protect yourself. So I found it difficult because you don’t really know how to say it, not to hurt their feelings and hurt your feelings at the same time...”

These findings are consistent with the literature that women are concerned with the reactions of their male partners, which impact women’s abilities to discuss future condom use [6]. Otto-Salaj and colleagues found that the mere act of suggesting a condom produced a variety of responses from heterosexual males [31]. Further exploratory analyses of men’s reactions are needed to understand the underlying mechanisms behind their concerns and to assist women with appropriate responses. The actual timing, such as the initial phase of dating, is an imperative predicator for the type of sexual behavior relationship involving safe prevention, consistent condom use and fidelity. Researchers suggest that women’s level of self-efficacy of condom use and negotiation strategies seem to determine whether some type of communication dialogue with sexual partners will be initiated as well as condom use consistency [32,33].

### 3.5. Condom Responsibility

A majority of participants felt that purchasing and having access to condoms was the male’s responsibility. Participants voiced the following views.

“I’m like it should be the guy’s responsibility to get the condom”.

“I mean as women we probably should keep condoms with us but I know me as well as most of my friends like we just expect the guy to have them since he’s the one that has to wear them we expect him to be the one to have them.”

Furthermore, women’s refusal to engage in sexual intercourse without a condom reinforced the male’s understanding of his role as illustrated below.

“Well my boyfriend tried that one time he was like, ‘Oh well I don’t have one’,

I was like, ‘Oh okay, see you tomorrow’, like he never forgot it again.”

However, some women felt that young women should be prepared in case their partner did not have a condom at the time of sexual intercourse.

“My mother told me if he doesn’t have one, you should. You should always be prepared, because you never know.”

“I feel like, because as a woman, you can’t just depend on a man to have a condom ready, like yes, it’s going on him, but at the end of the day, like if something goes wrong, it’s going to be on you and possibly him, not so much him, because it’s your body that you allowed it to happen to, so you should take responsibility.”

As women discussed their role, there appeared to be a double standard regarding women who carry condoms as evidenced in the literature [34]. Several women discussed the social stigma of women having condoms and the possibility of having a condom affecting their social character. Participants shared

“When I see like females picking up condoms it’s like weird because I know for me I don’t tend to initiate sex; I’m like it should be the guy’s responsibility to get the condom”.

“I do think there is, um, a negative connotation with females who carry condoms with them. And then that’s where like I don’t feel like it’s right. I don’t, but I don’t know, it’s just kind of like—I know in society it just makes it seem like the female, like that means like you’re ready to get down like whatever. So I don’t know. I do think both people are responsible, like if you want to have protected sex then both of you are responsible.”

The double standard evidence in the study may place women at greater risk than men because it may influence women’s sexual health behaviors in purchasing or securing condoms that would protect them against STIs [35].

## 4. Conclusions

AA college women in the current study described their involvement in taking an active and assertive role in condom discussions and negotiations. On the other hand, less than one third of the women in the current study reported consistent condom use. Strategies overall appear to be limited in scope and women articulated the need to counteract men’s responses. Findings from this study highlight the need to decrease women’s reliance on men to supply condoms and reduce stigma associated with female preparedness. There were a number of limitations associated with this study. The first limitation was the cross-sectional study design used for quantitative analysis. Moreover, the themes that emerged from the sample as a whole may differ by condom group. The current study did not link participants’ quantitative and qualitative data. Therefore it is not possible to determine if certain themes were more common in one condom group over another. Condom group differences should be examined in future qualitative studies of AA college women by stratifying focus groups based on condom use. Next, the findings for this study may not be generalizable to other HBCUs as the campus culture and environment may differ. Additionally, pregnancy prevention as a negotiation strategy was not included. Lastly, substance use including alcohol and illicit drug use, which are known to impact condom use, were not examined. Despite these limitations, this study provides exploratory data to address the gap in the literature about the relationship between condom negotiation strategies and frequency of condom use as well as provides a framework to better understand the context of condom negotiations among AA college women. Sexual disparities continue to exist within ethnicity, sex, and age groups among the college population. This study’s findings reveal possible factors that may increase and decrease condom use among college women. These factors should be further investigated for moderating and mediating influences. Further research is warranted to examine the role that education plays in STI prevention among African American female college students and the role higher education can play in bringing about behavioral change as a complement to public health interventions.

## References

[1] Centers for Disease Control and Prevention (2014). Sexually Transmitted Disease Surveillance 2013.

[2] Satterwhite C.L., Torrone E., Meites E., Dunne E.F., Mahajan R., Ocfemia M.C., Su J., Xu F., Weinstock H. (2013). Sexually transmitted infections among US women and men: Prevalence and incidence estimates, 2008. Sex. Transm. Dis..

[3] Lewis J.E., Miguez-Burbano M.J., Malow R.M. (2009). HIV risk behavior among college students in the United States. Coll. Stud. J..

[4] Milhausen R.R., McKay A., Graham C.A., Crosby R.A., Yarber W.L., Sanders S.A. (2013). Prevalence and predictors of condom use in a national sample of Canadian university students. Can. J. Hum. Sex..

[5] Holland K.J., French S.E. (2012). Condom negotiation strategy use and effectiveness among college students. J. Sex Res..

[6] Ferguson I.O., Quinn S.C., Beni E., Sandelowski M. (2006). The gender ratio imbalance and its relationship to risk of HIV/AIDS among African American women at historically black colleges and universities. AIDS Care.

[7] Hou S.I. (2009). HIV-related behaviors among black students attending Historically Black Colleges and Universities *versus* white students attending a traditionally white institution. AIDS Care.

[8] Walsh J., Fielder R., Carey K., Carey M. (2013). Changes in Women’s Condom Use over the First Year of College. J. Sex Res..

[9] Oswalt S., Wyatt T. (2013). Sexual health behaviors and sexual orientation in a U.S. national sample of college students. Arch. Sex. Behav..

[10] Fehr S.A., Vidourek R.A., King K.A. (2015). Intra- and Inter-personal Barriers to Condom Use among College Students: A Review of the Literature. Sex. Cult..

[11] Lewis L.M., Melton R.S., Succop P.A., Rosenthal S.L. (2000). Factors influencing condom use and STD acquisition among African American college women. J. Am. Coll. Health.

[12] Braithwaite K., Thomas V. (2001). HIV/AIDS knowledge, attitudes and risk-behaviors among African American and Caribbean college women. Int. J. Adv. Couns..

[13] Adepoju J.A., Watkins M.P., Richardson A.M. (2007). A quick survey of and HBCU’s first year nursing students’ perception of the HIV/AIDS phenomenon. JNBNA.

[14] D’Urso J., Thompson-Robinson M., Chandler S. (2007). HPV Knowledge and Behaviors of Black College Students at a Historically Black University. J. Am. Coll. Health.

[15] Adepoju J.A., Watkins M.P., Richardson A.M. (2009). A survey of a HBCU’s senior year nursing students’ perception of the HIV/AIDS phenomenon: A follow-up study. JNBNA.

[16] Jenkins C.C., Kennedy B.R. (2013). Exploratory study of sexual assertiveness and characteristics of African American women in negotiating condom use at an HBCU. J. Cult. Divers..

[17] Jemmott L.S., Jemmott J.B. (1991). Applying the theory of reasoned action to AIDS risk behavior: Condom use among black women. Nurs. Res..

[18] Noar S.M., Morokoff P.J., Harlow L.L. (2004). Condom negotiation strategies in a community sample of ethnically diverse sample of men and women. J. Appl. Soc. Psychol..

[19] Creswell J.W. (2007). Qualitative Inquiry & Research Design.

[20] DePaulo P. (2000). Sample Size in Qualitative Research. Quirks Marketing Research Media. Qualitative Research Issue. http://www.quirks.com/articles/a2000/20001202.aspx?searchID=383113850.

[21] Jarrett R.L., Morgan D.L. (1993). Focus group interviewing with low-income minority population: A research experience. Successful Focus Groups: Advancing the State of the Art.

[22] Noar S.M., Morokoff P.J., Harlow L.L. (2002). Condom negotiation in heterosexually active men and women. Development and validation of a condom influence strategy questionnaire. Psychol. Health.

[23] Sue D.W., Lin A.I., Torino G.C., Capodilupo C.M., Rivera D.P. (2009). Racial Microaggressions and Difficult Dialogues on Race in the Classroom. Cult. Divers. Ethn. Minor. Psychol..

[24] Madriz E., Denzin N.K., Lincoln Y.S. (2000). Focus groups in feminist research. Handbook of Qualitative Research.

[25] Braun V., Clarke V. (2006). Using thematic analysis in psychology. Qual. Res. Psychol..

[26] Sandelowski M. (1986). The problem of rigour in qualitative research. Adv. Nurs. Sci..

[27] Valentine P.A., Wright D.L., Henley G.L. (2003). Patterns of safer sex practices among allied health students at historically black colleges and universities. J. Allied Health.

[28] Hayes B.D., Holliday R.C., Wade B.H., Trawick C., Hodge M., Caplan L., Younge S., Quarshie A., Satcher D. (2009). A comprehensive examination of the health knowledge, attitudes and behaviors of students attending historically black colleges and universities. J. Health Care Poor Underserved.

[29] Bazargan M., Kelly E.M., Stein J.A., Husaini B.A., Bazargan S.H. (2000). Correlates of HIV risk-taking behaviors among African American College student: The effect of HIV knowledge, motivation, and behavioral skills. J. Natl. Med. Assoc..

[30] Rountree M., Mulraney M. (2010). HIV/AIDS Risk reduction intervention for women who have experienced intimate partner violence. Clin. Soc. Work J..

[31] Otto-Salaj L.L., Traxel N., Brondino M.J., Reed B., Gore-Felton C., Kelly J.A., Stevenson L.Y. (2010). Reactions of Heterosexual African-American Men to Women’s Condom Negotiation Strategies. J. Sex Res..

[32] Noar S.M., Carlye K., Cole C. (2006). Why communication is crucial: Meta-analysis of the relationship between safer sexual communication and condom use. J. Health Commun..

[33] Crosby R.A., DiClemente R.J., Salazar L.F., Wingood G.M., McDermott-Sales J., Young A.M., Rose E. (2013). Predictors of Consistent Condom Use among Young African American Women. AIDS Behav..

[34] Hynie M., Lydon J.E. (1995). Women’s perceptions of female contraceptive behavior: Experimental evidence of the sexual double Standard. Psychol. Women Q..

[35] Young M., Penhollow T., Bailey W. (2010). Hooking-up and condom provision: Is there a double standard?. Am. J. Health Stud..

